# Supplementary material for: Theory of Noise-Scaled Stability Bounds and Entanglement Rate Maximization in the Quantum Internet
Source: Sci Rep. 2020 Feb 17;10:2745. doi: 10.1038/s41598-020-58200-6 (PMC7026176; doi:10.1038/s41598-020-58200-6)
Supplement: Supplementary file 1 — Supplemental Information. [file 41598_2020_58200_MOESM1_ESM.pdf]

Theory of Noise-Scaled Stability Bounds and Entanglement Rate Maximization in the Quantum Internet

Laszlo Gyongyosi[1,2,3,*] **and Sandor Imre**[2]

[1]School of Electronics and Computer Science, University of Southampton, Southampton, SO17 1BJ, UK
[2]Department of Networked Systems and Services, Budapest University of Technology and Economics, Budapest, H-1117 Hungary
[3]MTA-BME Information Systems Research Group, Hungarian Academy of Sciences, Budapest, H-1051 Hungary
[*]l.gyongyosi@soton.ac.uk

ABSTRACT

Crucial problems of the quantum Internet are the derivation of stability properties of quantum repeaters and theory of entanglement rate maximization in an entangled network structure. The stability property of a quantum repeater entails that all incoming density matrices can be swapped with a target density matrix. The strong stability of a quantum repeater implies stable entanglement swapping with the boundness of stored density matrices in the quantum memory and the boundness of delays. Here, a theoretical framework of noise-scaled stability analysis and entanglement rate maximization is conceived for the quantum Internet. We define the entanglement swapping set that models the status of quantum memory of a quantum repeater with the stored density matrices. We determine the optimal entanglement swapping method that maximizes the entanglement rate of the quantum repeaters at the different entanglement swapping sets as function of the noise of the local memory and local operations. We prove the stability properties for non-complete entanglement swapping sets, complete entanglement swapping sets and perfect entanglement swapping sets. We prove the entanglement rates for the different entanglement swapping sets and noise levels. The results can be applied to the experimental quantum Internet.

A Appendix

A.1 Notations

The notations of the manuscript are summarized in Table A.1.

Table A.1. Summary of notations.

Notation	*Description*
N	An entangled quantum network, $N = (V, E)$, where V is a set of nodes, E is a set of entangled connections.
A	A source user (quantum node) in the quantum network.
B	A destination user (quantum node).
R_j	A current j-th quantum repeater, $j = 1, \ldots, q$, where q is the total number of quantum repeaters.
R_i	A previous neighbor of R_i.
R_k	A next neighbor of R_k (towards destination).
l	Level of entanglement.
$\mathrm{L}_l(x,y)$	An l-level entangled connection between quantum nodes x and y.
$d(x,y)_{\mathrm{L}_l}$	Hop-distance at an L_l-level entangled connection between quantum nodes x and y, $d(x,y)_{\mathrm{L}_l} = 2^{l-1}$.
O_C	An oscillator with frequency f_C, $f_C = 1/t_C$, serves as a reference clock.
C	A cycle, with $t_C = 1/f_C$.
π_S	An entanglement swapping period, in which the set $\mathscr{S}_I(R_j)$ of density matrices are swapped via the U_S entanglement swapping operator with the $\mathscr{S}_O(R_j)$ of density matrices, defined as $\pi_S = xt_C$, where x is the number of C.
π'_S	A next entanglement swapping period after π_S.
q	Total number of quantum repeaters in an entangled path $\mathscr{P}(A_i \to B_i)$, $q = d(A,B)_{\mathrm{L}_l} - 1$.
B_F	Entanglement throughput [Bell states per π_S].
$\lvert B_F \rvert$	Number of entangled states [Number of Bell states].
$\mathrm{L}_l(k)$	A k-th entangled connection.
$B_F(\mathrm{L}_l(k))$	Entanglement throughput of the entangled connection $\mathrm{L}_l(k)$ [Bell states per π_S].
ρ	In a j-th quantum repeater R_j, an ρ incoming density matrix is a half of an entangled state $\lvert\beta_{00}\rangle$ received from a previous neighbor node R_{j-1}.
σ	The σ outgoing density matrix in R_j is a half of an entangled state $\lvert\beta_{00}\rangle$ shared with a next neighbor node R_{j+1}.
U_S	Entanglement swapping operation is a local transformation that swaps an incoming density matrix ρ with an outgoing density matrix σ in a quantum repeater R.
$\mathscr{S}_I(R_j)$	Set of incoming density matrices stored in the quantum memory of R_j, $\mathscr{S}_I(R_j) = \bigcup_i \rho_i$, where ρ_i is an i-th density matrix.
$\mathscr{S}_O(R_j)$	Set of outgoing density matrices stored in the quantum memory of R_j, $\mathscr{S}_O(R_j) = \bigcup_i \sigma_i$, where σ_i is an i-th density matrix.

$\mathscr{S}_I^*(R_j)$	A complete set of incoming density matrices. Set $\mathscr{S}_I(R_j)$ formulates a $\mathscr{S}_I^*(R_j)$ complete set if $\mathscr{S}_I(R_j)$ contains all the $Q=\sum_{i=1}^{N}\lvert B_i\rvert$ incoming density matrices per π_S that is received by R_j in a swapping period, where N is the number of input entangled connections of R_j, $\lvert B_i\rvert$ is the number of incoming densities of the i-th input connection per π_S, thus $\mathscr{S}_I(R_j)=\bigcup_{i=1}^{Q}\rho_i$ and $\lvert\mathscr{S}_I(R_j)\rvert=Q$.
$\mathscr{S}_O^*(R_j)$	A complete set of outgoing density matrices. An $\mathscr{S}_O(R_j)$ set formulates a $\mathscr{S}_O^*(R_j)$ complete set, if $\mathscr{S}_O(R_j)$ contains all the N outgoing density matrices that is shared by R_j during a swapping period π_S, thus $\mathscr{S}_O(R_j)=\bigcup_{i=1}^{N}\sigma_i$ and $\lvert\mathscr{S}_O(R_j)\rvert=N$.
$\mathscr{S}(R_j)$	An entanglement swapping set of R_j, $\mathscr{S}(R_j)=\mathscr{S}_I(R_j)\bigcup\mathscr{S}_O(R_j)$ that describes the status of the quantum memory in R_j.
$\mathscr{S}^*(R_j)$	A complete entanglement swapping set. A $\mathscr{S}(R_j)$ is a $\mathscr{S}^*(R_j)$ complete swapping set, if $\mathscr{S}^*(R_j)=\mathscr{S}_I^*(R_j)\bigcup\mathscr{S}_O^*(R_j)$, with cardinality $\lvert\mathscr{S}^*(R_j)\rvert=Q+N$.
$\mathscr{S}^*(R_j)$	A perfect entanglement swapping set. A $\mathscr{S}^*(R_j)$ complete swapping set is a $\hat{\mathscr{S}}(R_j)=\hat{\mathscr{S}}_I(R_j)\bigcup\hat{\mathscr{S}}_O(R_j)$ perfect swapping set at a given π_S, if $\lvert\hat{\mathscr{S}}(R_j)\rvert=N+N$.
$\mathscr{S}_{R_j}^{(\pi_S)}((R_i,\sigma_k))$	A coincidence set, a subset of incoming density matrices in $\mathscr{S}_I(R_j)$ of R_j received from R_i that requires the same outgoing density matrix σ_k from $\mathscr{S}_O(R_j)$ for the entanglement swapping.
$Z_{R_j}^{(\pi_S')}((R_i,\sigma_k))$	Cardinality of the coincidence set $\mathscr{S}_{R_j}^{(\pi_S)}((R_i,\sigma_k))$ [Number of Bell states].
$\lvert B(R_i(\pi_S),\sigma_k)\rvert$	Number of density matrices arrive from R_i to R_j for swapping with σ_k at π_S. It increments the cardinality of the coincidence set as $Z_{R_j}^{(\pi_S')}((R_i,\sigma_k))=Z_{R_j}^{(\pi_S)}((R_i,\sigma_k))+\lvert B(R_i(\pi_S),\sigma_k)\rvert$, where π_S' is a next entanglement swapping period [Number of Bell states].
$\lvert B_{R_i}(\pi_S)\rvert$	Incoming entanglement rate of R_j per a π_S, defined as $\lvert B_{R_j}(\pi_S)\rvert=\sum_{i,k}\lvert B(R_i(\pi_S),\sigma_k)\rvert$, where $\lvert B(R_i(\pi_S),\sigma_k)\rvert$ refer to the number of density matrices arrive from R_i for swapping with σ_k per π_S [Bell states per π_S].
$D(\pi_S)$	Delay, measured in entanglement swapping periods π_S [Number of π_S periods].
$\lvert B'_{R_j}(\pi_S)\rvert$	Outgoing entanglement rate of R_j, defined as $\lvert B'_{R_j}(\pi_S)\rvert=\left(1-\frac{L}{N}\right)\frac{1}{1+D(\pi_S)}\left(\lvert B_{R_j}(\pi_S)\rvert\right)$, where L is the loss, $0<L\leq N$ [Bell states per π_S].
$\zeta(\pi_S)$	Entanglement swapping procedure at a given π_S.
$\mathscr{S}_I^{(\pi_S)}(R_j)$	Set of incoming densities of R_j at π_S.
$\lvert\mathscr{S}_I^{(\pi_S)}(R_j)\rvert$	Cardinality of the set $\mathscr{S}_I^{(\pi_S)}(R_j)$ [Number of Bell states].
γ	Noise coefficient, models the noise of the local quantum memory and the local operations, $0\leq\gamma\leq 1$.
N	Number of coincidence sets of R_j, and number of outgoing connections of R_j.
L	Number of losses, $0\leq L\leq N$.
M	Reduced number of swapped incoming and outgoing density matrices per π_S at L losses, $M=N-L$.
$\gamma(\pi_S)$	Noise at a given π_S.
$Z_{R_j}(\pi_S)$	A matrix of all coincidence set cardinalities for all input and output connections at π_S, defined as $Z_{R_j}(\pi_S)=Z_{R_j}^{(\pi_S)}((R_i,\sigma_k))_{i\leq N,k\leq N}$.

$\omega(\pi_S)$	Weight coefficient, for a given entanglement swapping $\zeta(\pi_S)$ at a given π_S is as $\omega(\pi_S) = \sum_{i,k} \zeta_{ik}(\rho_A, \sigma_k) Z_{R_j}^{(\pi_S)}((R_i, \sigma_k)) = \langle \zeta(\pi_S), Z_{R_j}(\pi_S) \rangle$, where $\langle \cdot \rangle$ is the inner product.
$\omega^*(\pi_S)$	Maximized weight coefficient.
$\zeta^*(\pi_S)$	Optimal entanglement swapping method at $\gamma(\pi_S) = 0$
$\lvert\chi(\pi_S)\rvert$	Norm, defined for an entanglement swapping $\chi(\pi_S)$.
$\mathscr{L}(Z_{R_j}(\pi_S))$	Lyapunov function of $Z_{R_j}(\pi_S)$, as $\mathscr{L}(Z_{R_j}(\pi_S)) = \sum_{i,k}\left(Z_{R_j}^{(\pi_S)}((R_i, \sigma_k))\right)^2$.
C_1,C_2	Constants, $C_1 > 0$, $C_2 > 0$.
$\Delta_{\mathscr{L}}$	Difference of Lyapunov functions $\mathscr{L}(Z_{R_j}(\pi'_S))$ and $\mathscr{L}(Z_{R_j}(\pi_S))$, where π'_S is a next entanglement swapping period, defined as $\Delta_{\mathscr{L}} = \mathscr{L}(Z_{R_j}(\pi'_S)) - \mathscr{L}(Z_{R_j}(\pi_S))$.
$\lvert\bar{B}(R_i(\pi'_S), \sigma_k)\rvert$	A normalized number of arrival density matrices, $\lvert\bar{B}(R_i(\pi'_S), \sigma_k)\rvert \leq 1$, from R_i for swapping with σ_k at a next entanglement swapping period π'_S, defined as $\lvert\bar{B}(R_i(\pi'_S), \sigma_k)\rvert = \frac{\lvert B(R_i(\pi'_S), \sigma_k)\rvert}{\lvert B_{R_j}(\pi_S)\rvert}$, where $\lvert B_{R_j}(\pi_S)\rvert = \sum_{i,k} \lvert B(R_i(\pi_S), \sigma_k)\rvert$ is a total number of incoming density matrices of R_j from the N quantum repeaters.
$\mathbb{E}(\lvert\bar{B}(R_i(\pi_S), \sigma_k)\rvert)$	Expected normalized number of density matrices arrive from R_i for swapping with σ_k at π_S.
α_{ik}	Parameter, defined as $\alpha_{ik} = Z_{R_j}^{(\pi_S)}((R_i, \sigma_k)) \bar{B}(R_i(\pi_S), \sigma_k)$.
ν_z	A constant, $\nu_z \geq 0$.
C_1	Constant, $C_1 = 1 - \sum_z \nu_z$.
$\lvert Z_{R_j}(\pi_S)\rvert$	Cardinality of the coincidence sets at a given π_S, as $\lvert Z_{R_j}(\pi_S)\rvert = \sum_{i,k} Z_{R_j}^{(\pi_S)}((R_i, \sigma_k)) = \lvert \mathscr{S}_I(R_j)\rvert$.
$\lvert B_{R_i}(\pi_S)\rvert$	Total number of incoming density matrices in R_j per a given π_S, as $\lvert B_{R_j}(\pi_S)\rvert = \sum_{i,k} \lvert B(R_i(\pi_S), \sigma_k)\rvert$.
$\tilde{\pi}_S$	An extended entanglement swapping period, defined as $\tilde{\pi}_S = \pi_S + D(\pi_S)$, with $\pi_S / (\tilde{\pi}_S) \leq 1$ [Number of π_S periods].
$B'_{R_j}(\pi_S)$	Outgoing entanglement rate per π_S for a particular entanglement swapping set [Bell states per π_S].
$f(\cdot)$	Sub-linear function.
$\xi(\gamma)$	Parameter, defined as $\xi(\gamma) = \frac{(N-L)}{2C_1} f(\gamma(\pi_S))$.
β	Parameter, defined as $\beta = \sum_{i,k}\left(\lvert\bar{B}(R_i(\pi_S), \sigma_k)\rvert - \lvert\bar{B}(R_i(\pi_S), \sigma_k)\rvert^2\right)$, where $\lvert\bar{B}(R_i(\pi_S), \sigma_k)\rvert$ refers to the normalized number of density matrices arrive from R_i for swapping with σ_k at π_S as $\lvert\bar{B}(R_i(\pi_S), \sigma_k)\rvert = \frac{\lvert B(R_i(\pi_S), \sigma_k)\rvert}{\sum_i \lvert B(R_i(\pi_S), \sigma_k)\rvert}$.
P	Number of entanglement swapping periods .
$D(\pi_S)$	Delay per π_S at a non-complete entanglement swapping set.

$D^*(\pi_S)$	Delay per π_S at a complete entanglement swapping set.
$\hat{D}(\pi_S)$	Delay per π_S at a perfect entanglement swapping set.
$\left\lvert Z_{R_j}(\pi_S) \right\rvert$	Cardinality of the coincidence sets at a given π_S, for a non-complete entanglement swapping set.
$\left\lvert Z^*_{R_j}(\pi_S) \right\rvert$	Cardinality of the coincidence sets at a given π_S, for a complete entanglement swapping set.
$\left\lvert \hat{Z}_{R_j}(\pi_S) \right\rvert$	Cardinality of the coincidence sets at a given π_S, for a perfect entanglement swapping set.
π^*_S	An extended entanglement swapping period, defined as $\pi^*_S = (1+h)\,\pi_S$, where $h > 0$ [Number of π_S periods].
